# Selection and Characterization of Packaged FBG Sensors for Offshore Applications

**DOI:** 10.3390/s18113963

**Published:** 2018-11-15

**Authors:** Lei Wu, Muneesh Maheshwari, Yaowen Yang, Wensheng Xiao

**Affiliations:** 1 School of Civil and Environmental Engineering, Nanyang Technological University, Singapore 639798, Singapore; wuleiupc@ntu.edu.sg (L.W.); muneesh@ntu.edu.sg (M.M.); 2 Maritime Institute, Nanyang Technological University, Singapore 639798, Singapore; 3 College of Mechanical and Electronic Engineering, China University of Petroleum, Qingdao 266580, China; xiaows@upc.edu.cn

**Keywords:** structural health monitoring, offshore structure, FBG sensor, FPSO

## Abstract

With the development in the exploitation of maritime resources, the structural health monitoring (SHM) of offshore structures becomes necessary. This study focuses on addressing the practical issues of application of fiber Bragg grating (FBG) sensors for the SHM of offshore structures, in particular an FPSO (floating, production, storage, and offloading unit) vessel. Due to the harsh marine environment and tough working conditions, the FBG sensors must have sufficient protection and good repeatability for long-term monitoring. Thorough research has been conducted to identify the most suitable, commercially available protection packaging for FBG sensors for offshore applications. Further, the performance of the selected FBG sensor packaging is tested under conditions of strong sunlight, heavy rain, and salty water in order to emulate the marine environment. Moreover, the installation method of the packaged FBG sensors is equally important, as it ensures the repeatability and durability of the sensors for their long-term performance. It is shown that the packaged FBG sensors can be installed using resin-based epoxy to maintain the repeatability of the sensor over the long-term. Further, the packaged FBG sensors are installed and tested on a simple FPSO model. The experimental results under full load and ballast draft conditions show that the proposed FBG sensors are competent for the SHM of offshore structures.

## 1. Introduction

Structural health monitoring (SHM) refers to detecting and analyzing the changes in structures, and indicating damage and property degradation [1]. The SHM system, which detects the changes of various parameters including strain, vibration, temperature, and so on, can help evaluate the design assumptions and parameters for existing structures, as well as provide relevant information to improve the design specifications for future similar structures [2,3]. Therefore, SHM has been applied in various industries [4,5], such as building construction [6], mechanical and aerospace structures, etc. In recent years, the exploitation of maritime resources has developed rapidly. Usually, the offshore structures are built for the production and transmission of electricity, oil, gas, and other resources. Due to the complexity and high cost of offshore structures, it is indispensable to monitor their health state in the marine environment [7,8]. An FPSO (floating, production, storage, and offloading unit) is a floating vessel used by the offshore oil and gas industry for the production and processing of hydrocarbons, and could be in service in remote and deep waters for a continuous period of 20 years [9,10]. Structural damage in the FPSO can lead to a halt in production and heavy subsequent financial losses; even personnel safety can be threatened. Therefore, the offshore oil and gas industry needs a very reliable and effective SHM system. The prerequisite for SHM technology to be used on offshore structures is that it must be non-intrusive, robust, and perform with a high degree of repeatability over the long term, as most of the offshore structures remain operational for a long period of time in a very harsh environment.

In general, a typical SHM system consists of three parts: sensors, a data transmission system, and a health evaluation system. The structural responses are first measured by the sensors; then, they are transmitted through the data transmission system, and finally analyzed by the health evaluation system [2]. The accuracy and reliability of the health-condition evaluation results largely depend on the variety, quantity, and quality of the monitoring data that is acquired from the sensors. Actually, the traditional sensors such as strain gauges, piezoelectric sensors, etc. have played leading roles in the field of SHM [11,12]. In recent decades, as a new and fast-growing method, fiber Bragg grating (FBG) sensors, have been very popular and advantageous for the health monitoring of different structures. Compared with the traditional sensors, FBG sensors have significant advantages, including their immunity to electromagnetic interference, electric passivity, light weight, multiplexing capabilities, and durability [13,14]. FBG sensors are completely non-intrusive, highly accurate, and repeatable. However, one of the main drawbacks of FBG sensors is the high cost of the interrogator compared with other traditional sensing systems. In recent years, researchers have investigated the application of FBG sensors on the offshore structures [15].

Mieloszyk and Ostachowicz presented an application of FBG sensors for the SHM of an offshore wind turbine support structure model; the experimental investigation verified the usefulness of FBG sensors as a part of the system permanently installed on the underwater elements of an offshore structure [16]. Based on the strain measurements obtained by a network of FBG sensors, a method for the detection and localization of the damage in a wind turbine model was proposed by Opoka et al. [17]. Yi carried out a series of experiments to detect the local damage for jacket-type offshore structures using FBG sensors [18]. Xu et al. introduced a new FBG-based bundle-structure riser stress-monitoring sensor that obtained the continuous data of an ocean oil-drilling platform; the experimental results show that the sensor can work well under water [19]. Majewska et al. used FBG sensors to determine the strain/stress level of the foremast of a sailing ship that was surrounded by a harsh marine environment and exposed to long-term cyclic loadings [20]. Ren et al. encapsulated 56 FBG sensors in stainless-steel tubes to measure the vortex-induced vibrations in a 28-meter model pipeline, and the tests showed that the FBG sensors had good sensitivity and were able to sustain a large amount of loading cycles without suffering from damage [21]. Moreover, Sun et al. used FBG sensors for the measurement of the dynamic stress of a ship thruster inner skin [22]. FBG sensors were used to compose a monitoring system for the soft yoke mooring system of an FPSO [23]. Li et al. provided strain-based damage characterization techniques using FBG sensors for the health monitoring of composite maritime structural joints [24]. Ren et al. described a demonstration of FBG sensor application in offshore platform monitoring [25]. Kim et al. used two kinds of sensors—piezoceramic and fiber optic transducers—to monitor the structural health of large welded structures [26].

Since a bare FBG sensor is quite fragile and easily damaged, it requires sufficient protection to be used for field applications [27]. In fact, various FBG sensor protection schemes are available commercially [28]. The fully pasted FBG [29,30] packaging of the pre-stretched FBG with double-end fixed [31] metallic packaging [32,33] and a packaging tube [34] are the most commonly used methods for FBG protection [35]. Actually, the encapsulation of FBG has a significant influence on the FBG characteristics that directly affect the measurement accuracy, such as strain transfer, temperature characteristics, and spectral shape [36]. Therefore, the protection of FBG sensors remains important for offshore applications, and investigations on suitably packaged FBG sensors for offshore structures are necessary.

Generally, practical issues related to the offshore application of FBG sensors include the selection of sensor protection, installation method, measurement process, and responses of the sensor to harsh environments. Most of the current research studies have just focused on one aspect of offshore applications. In this study, a comprehensive investigation of these topics is conducted. Characteristics such as the robustness, sensitivity, stability, and repeatability of three commercially available packaged FBG sensors are studied in this work. Based on all of the characterization results, the composite embedded FBG sensors are recommended for offshore applications. The installation of packaged FBG sensors is another important issue, as it ensures the repeatability and durability of the sensors. Also, the installation method must be non-invasive for offshore applications. Usually, the packaged FBG sensors are installed by gluing, bolting, or welding. Although the methods of bolting and welding can fix the sensors firmly, the structural integrity of the monitored structures may be hampered. The composite embedded FBG sensors can be easily installed by gluing them on the structures using resin-based epoxy, which is a non-invasive method. Although the fatigue performance of the gluing method cannot be as good as that of the bolting or welding method, the results from the dynamic test show that resin-based epoxy works well with the composite embedded FBG sensors as far as the repeatability of the sensor is concerned. Further, the experimental results show that the repeatability of the composite embedded FBG sensors while being subjected to harsh environmental conditions such as strong sunlight, heavy rain, and salty water is maintained, which confirms the durability of the sensors. Further, an FBG monitoring system for offshore structures is introduced based on the selected FBG sensor. Finally, the FBG monitoring system is tested on an FPSO model, and the results show that the proposed system is competent for the SHM of offshore structures in terms of its practicability and accuracy.

## 2. Principle of FBG Sensor

**Figure 1:**
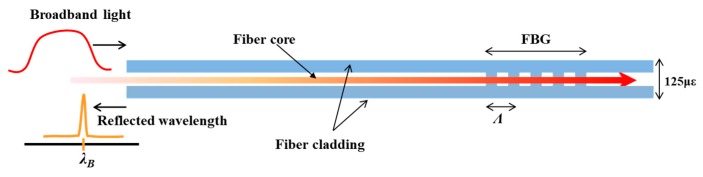
Wavelength filtering by fiber Bragg grating (FBG) sensor.

In FBG sensors, grating (periodic refractive index profile) is inscribed into the core of the fiber, as shown in Figure 1. An excimer laser (248 nm) and phase masks are used to inscribe this grating structure into the core of the fiber. If the light from a broadband source is launched into the FBG, a narrow wavelength peak (full-width half-maximum, FWHM ≈ 0.1–0.2 nm) is reflected back by the FBG, or in other words, an FBG works as a wavelength filter. This wavelength is called the Bragg wavelength (*λ_B_*). The Bragg wavelength changes as the grating period (the distance between the consecutive grooves) changes [37]. The Bragg wavelength peak shifts if the strain, temperature, pressure, etc. changes in the FBG. The measurement done with the FBG is highly accurate. The Bragg wavelength is given as:

(1) $$\lambda_{B}=2n_{eff}\Lambda$$

where *n_eff_* is the effective refractive index of the core of the fiber, and *Λ* is the grating period of the FBG. When the FBG is subjected to external perturbations, the grating period increases or decreases, and hence, the Bragg wavelength increases or decreases, respectively. The wavelengths’ drift ($\Delta \lambda_{B}$) induced by the temperature change (ΔT) and strain change (Δε) is given by:

(2) $$\frac{\Delta \lambda_{B}}{\lambda_{B}}=\kappa_{T}\Delta T+\kappa_{\varepsilon }\Delta \varepsilon$$

where *Δλ_B_* is the change in the Bragg wavelength; and $\kappa_{T}$ and $\kappa_{\varepsilon }$ are the temperature and strain sensitivity coefficients, respectively [8].

## 3. Characterization of Commercially Available Packaged/Protected FBG Sensors

**Figure 2:**
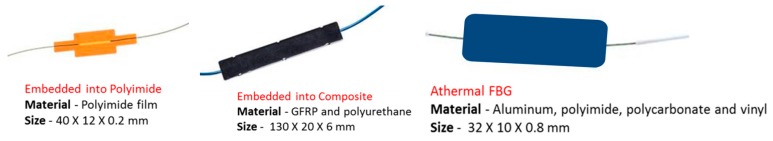
FBG sensors with various protection schemes.

In this section, experiments are conducted to find out the most suitable protection/packaging for FBG sensors that can be part of the SHM system for the offshore structures. In this research, three representative and competitive FBG protections (as shown in Figure 2), including an FBG sensor embedded into polyimide (protection material: polyimide film), an FBG sensor embedded into composite (protection material: glass-fiber-reinforced plastic and polyurethane), and an athermal FBG sensor (protection material: aluminum, polyimide, polycarbonate, and vinyl), are tested. For the athermal FBG sensor, the packaging consists of an assembly of materials with different coefficients of thermal expansion (CTE). Usually, one material has a high CTE and another has a low CTE; thereby, the temperature compensation can be realized [38].

### 3.1. Static Tests

For the purpose of verifying the credibility and sensitivity of the three FBG sensors, a bare FBG sensor (FBG length: 10 mm) and strain gauge are used for comparison, and the simulation data obtained by finite element analysis (FEA) software is illustrated as the theoretical values.

**Figure 3:**
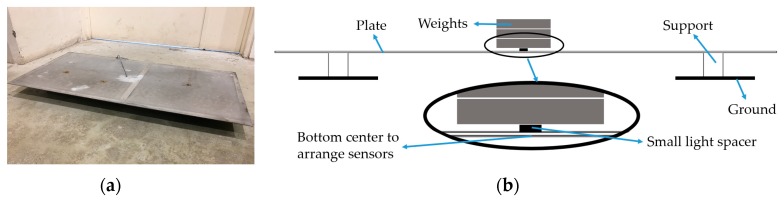
Experimental design of static tests. (**a**) Actual aluminum plate; (**b**) Schematic diagram of the tests.

**Figure 4:**
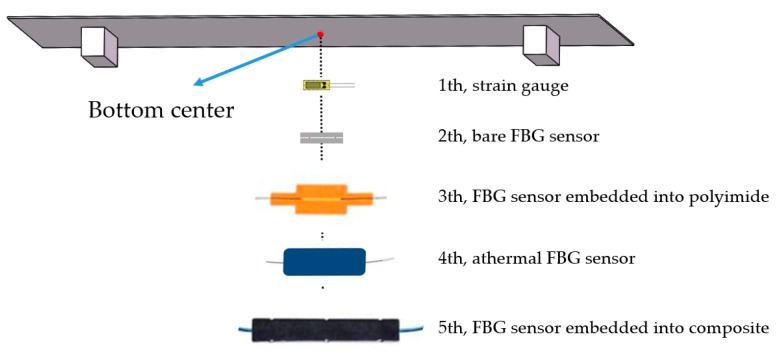
Schematic diagram illustrating the position and sequence when arranging sensors.

In static tests, all five sensors are used to measure the strain value of an aluminum plate of size 1030 mm × 530 mm × 3 mm under simply supported loading conditions, as shown in Figure 3. During the testing, all of the sensors (the strain gauge, the bare FBG sensor, and the three protected FBG sensors) are bonded to the center of the aluminum plate one by one (as shown in Figure 4). The sensors are bonded to the bottom surface of the plate using epoxy. Since the layer of epoxy that provides strong bonding between the FBG sensor and the metal is very thin, the effect of glue can be neglected. Before installing the next sensor, the previous one must be removed. The loading force at the center of the plate varies from 10 N to the maximum weight with 10 N increments. Considering that the average strain value on the FPSO main deck is around 200 με, the maximum loading force 60 N is selected, as it leads to the average strain level on the FPSO main deck. All of the tests are carried out in the laboratory, in which the temperature is relatively steady. Since the time duration for every test is short (about five minutes), the temperature can be considered constant during the test, and the changes of wavelength are only caused by the strain.

**Figure 5:**
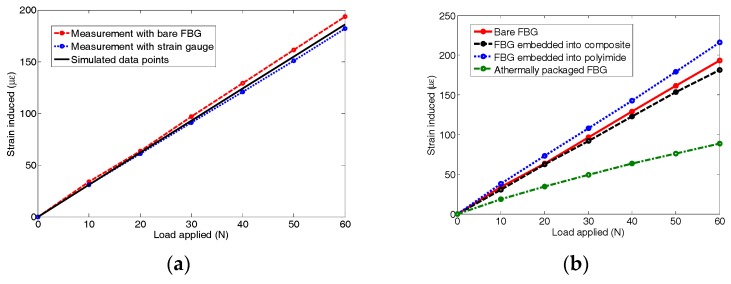
(**a**) Comparison of bare FBG sensors and strain gauge measurements with simulated data points; (**b**) Strain sensitivity comparison of protected FBG sensors with the bare FBG sensor.

**Figure 6:**
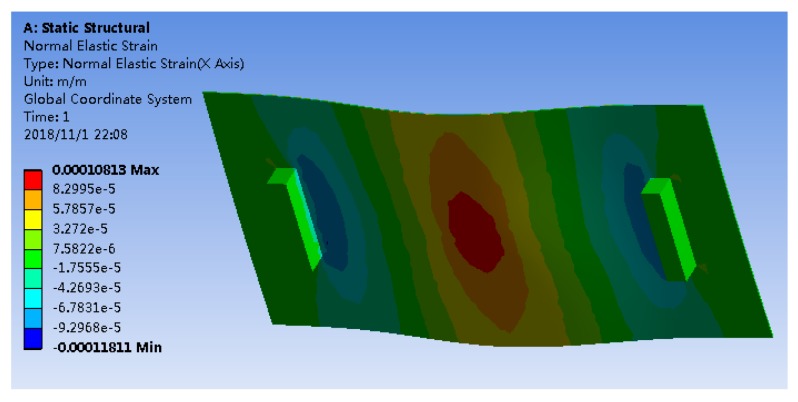
Normal strain distribution corresponding to 60 N from simulation.

The strain values measured by the five sensors under various loads are plotted in Figure 5, and compared with the simulated data from ANSYS. Table 1 shows all of the measured data from the sensors and the percentage differences compared with the simulation results. Moreover, the normal strain distribution of the plate corresponding to a load of 60 N is shown in Figure 6.

Clearly, similar to the strain gauge, the measurement data obtained by bare FBG sensor are very close to the simulated data, establishing the credibility of the bare FBG sensor. However, the bare FBG sensor cannot be used directly. Different protection materials have different influences on the sensitivity of the FBG sensor. As shown in Figure 5b, the results of the FBG sensor embedded into polyimide and the FBG sensor embedded into composite match well with the results of the bare FBG sensor, but not those obtained by the athermal FBG sensor. It is seen that the sensitivity of the FBG sensor is drastically reduced due to the athermal packaging, and the embedding of FBG sensors into polyimide or composite hardly hampers their sensitivity. In other words, the sensitivity of the FBG sensors embedded into polyimide and composite remains more or less the same as that of the bare FBG sensor. As shown in Table 1, the measurements of the FBG sensor embedded into composite are much closer to the simulated data than the measurements of the FBG sensor embedded into polyimide. Thus, the FBG sensor embedded into composite has a better performance than the other two sensors from the loading test.

The repeatability of sensors is equally important. The repeatability of the bare FBG sensor is certainly very high. For the packaged sensor, the protection layers may play a role affecting the repeatability. To ascertain such an effect, the loading tests are repeated six times with all the same sensors and the same plate, and the variance and standard deviation (Std) are calculated for each load. In Table 2, the maximum value of standard deviation for every customized FBG sensor is compared. It is evident that the FBG sensor embedded into the composite material is the most consistent (after the bare FBG sensor), indicating its best repeatability among all of the protected FBG sensors.

In summary, from results of the static tests, the FBG sensor embedded into composite is the best one among the three FBG sensors with protection material.

### 3.2. Dynamic Tests

**Figure 7:**
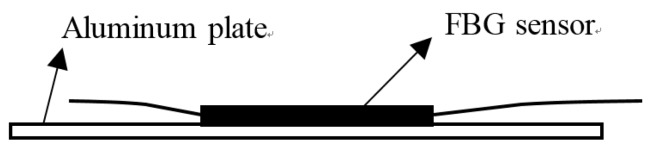
Schematic diagram when bonding the sensors on the aluminum plate.

**Figure 8:**
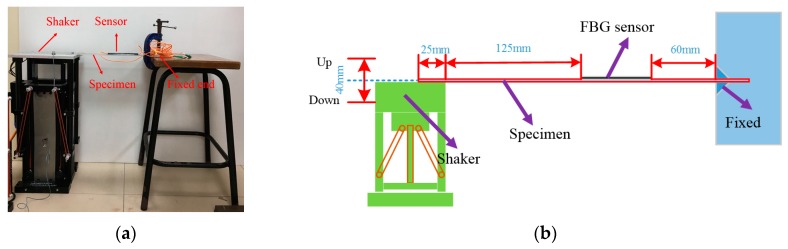
(**a**) Experimental setup of dynamic tests; (**b**) Schematic diagram of experimental setup.

Dynamic loads are very common in offshore applications, and dynamic tests for the protected FBG sensors must be conducted. Considering the static test results of the three FBG sensors, the most suitable one is utilized to carry out the dynamic tests. Thus, the FBG sensor embedded into composite that has the best performance in static tests is bonded to an aluminum plate with a size of 450 mm × 94 mm × 2 mm by using resin-based epoxy, as shown in Figure 7. Then, the specimen is fixed, as shown in Figure 8a. One end of the plate is clamped, and the other one is screwed to a vibration shaker. The shaker is fed with a sinusoidal signal of frequency of 1.0 Hz through a function generator. Hence, one end of the plate is fixed, and the other one is moving up and down at a frequency of 1.0 Hz with the shaker, inducing cyclic strain in the composite-embedded FBG sensor bonded to the other end. The detailed parameters of the experimental setup of dynamic tests are shown in Figure 8b.

**Figure 9:**
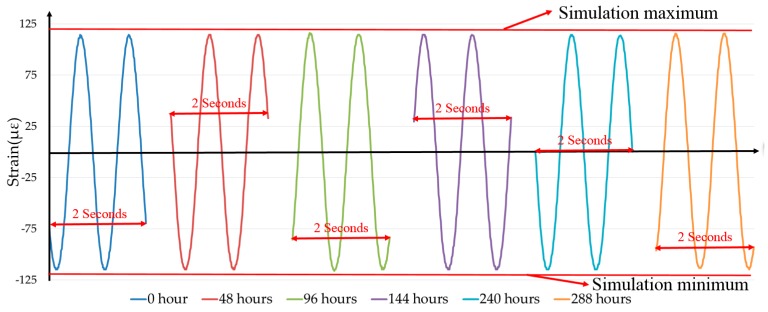
The dynamic strain measurement cycle samples at different time points.

In order to test the stability and repeatability of the sensor, the shaker runs continuously for about 12 days (12 × 24 = 288 h). The strain data samples (for two cycle periods) are collected at 0 h (beginning), 48 h, 96 h, 144 h, 240 h, and 288 h. The wavelength data is converted into the strain data and plotted in Figure 9. The temperature for a data cycle can be considered constant, as one data cycle takes only two seconds. The temperature might be different for data cycles taken at different times; however, the peak-to-peak values of the data cycles are independent of the temperature. Hence, the temperature information is irrelevant for the dynamic test. It can be seen that the peak-to-peak amplitude of the data cycle remains constant after 1.0368 × 10^6^ cycles (in 12 days), indicating the reliability and repeatability of the composite-embedded FBG sensors. The simulation results (the maximum and minimum of strain values) obtained from FEA based on the parameters shown in Figure 8b match well with the experimental data, as shown Figure 9. Thus, the composite-embedded FBG sensor repeats itself excellently in dynamic loading/unloading conditions.

Therefore, we can conclude that the FBG sensor embedded into composite is suitable for offshore applications in consideration of its credibility, sensitivity, stability, and repeatability.

### 3.3. Durability Tests

Due to the harsh environment, the offshore applications raise many extra requirements for the FBG sensors. The FBG sensors and their installation methods must be durable enough to work under some extreme conditions such as strong sunlight, heavy rain, high humidity, etc. Especially, they must work well in saline water. Since the seawater is oxygen-containing and conductive, the coating layer of the FBG sensor and the adhesive may fail after a long period of work [39]. In order to test the performance of the FBG sensor embedded into composite in the harsh environment, the specimen used in Section 3.2 is subjected to harsher environmental conditions for a period of 15 days. In detail, the specimen is put outside to endure sunlight, rain, and other weather conditions from 9:00 to 17:30 every day to simulate the real work conditions over 15 days of experimentation.

**Figure 10:**
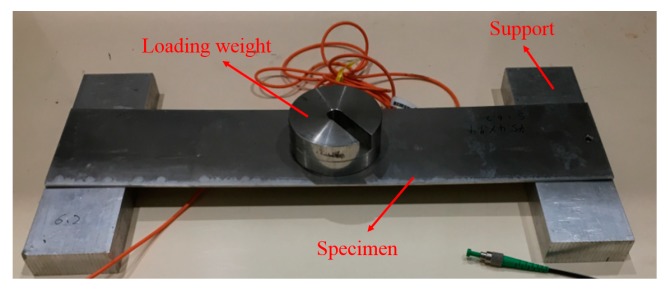
Experimental setup of loading tests.

Moreover, to verify the durability of the FBG sensor in sea water, the specimen was put into salty water that had a similar salinity (35‰) to sea water overnight (from 19:00 to 08:00) for 15 days. The loading test was conducted on the specimen every day at 8:45 after taking the specimen out of the saline water, after which the specimen was put in the outside weather conditions. This cycle of events continued for 15 days. In the loading test, 5-N, 10-N, 15-N, and 20-N weights were put at the center of the aluminum plate, as shown in Figure 10, and the responses of the FBG sensor were recorded.

**Figure 11:**
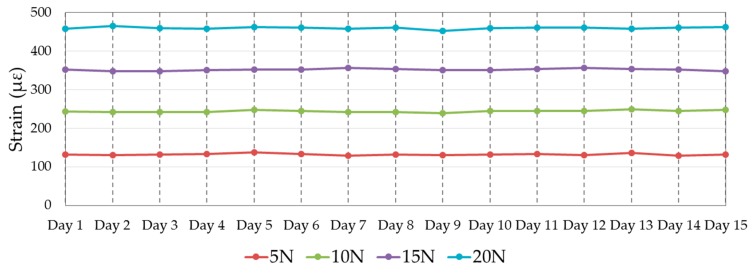
Strain values measured by the FBG sensor under different weights on different days.

Before conducting the loading tests, the specimen was placed in the laboratory for about half an hour for temperature stabilization. However, the temperature in the laboratory while conducting the loading test might be different on different days. This issue can be overcome by recording a reference value of the FBG sensor (Bragg wavelength) every day before conducting the loading test and temperature variations during the test can be conveniently considered negligible. As shown in Figure 11, the responses of the FBG sensor to the same load remain consistent over the 15 days. Actually, the biggest difference during the 15 days of experimentation was 10.71 με (between the strain value at 20 N on day 2 and the strain value at 20 N on day 9), which constitutes only a 2.36% error. In brief, the experimental results show that the FBG sensor embedded into composite work well in the harsh offshore environment, and the demonstrated fixing method for the FBG sensor can be adopted.

### 3.4 Calibration for Temperature Variations

**Figure 12:**
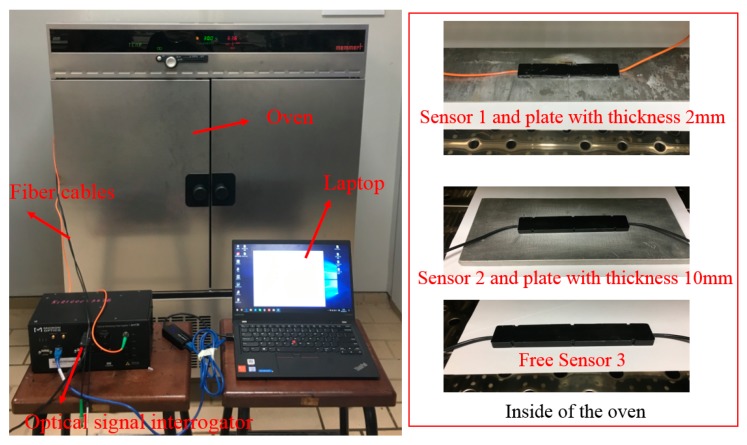
Experimental design for the calibration of temperature variations.

In an offshore environment, temperature variations might take place quite often. Therefore, the composite embedded FGB sensor must be characterized for temperature variations. In the experiment, three FBG sensors embedded into the composite are used. Two FBG sensors are attached to two separate plates of the same material that have different thicknesses. In detail, the first one (Sensor 1) is bonded to an aluminum plate with a size of 450 mm × 94 mm × 2 mm (the specimen used in Section 3.2 and Section 3.3), and the second one (Sensor 2) is bonded to an aluminum plate with a size of 200 mm × 110 mm × 10 mm. For comparison, the third one (Sensor 3) is free. All three FBG sensors were put into an oven at different temperatures ranging from 20 °C to 60 °C with a 5 ^°^C increment. The whole system for the experiments is shown in Figure 12.

**Figure 13:**
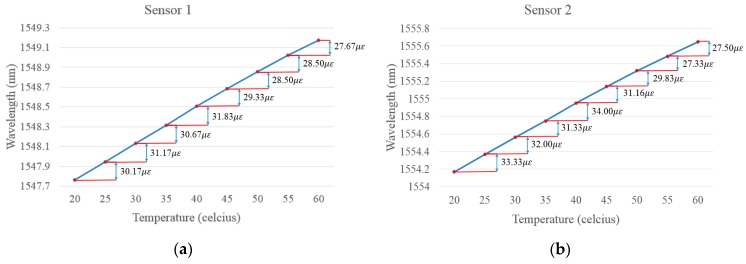

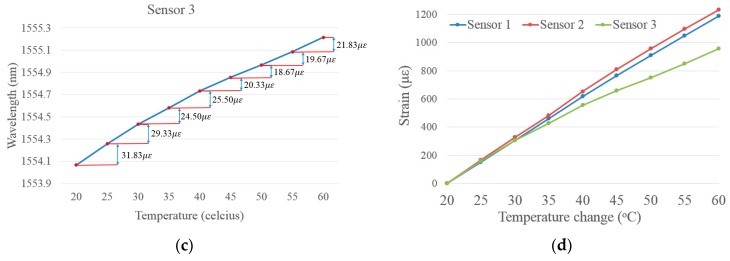
FBG sensors’ responses to temperature variations. (**a**) Temperature response of Sensor 1; (**b**) Temperature response of Sensor 2; (**c**) Temperature response of Sensor 3; (**d**) Comparison of the three FBG sensors.

The responses of the three FBG sensors to temperature variations are plotted in Figure 13. Clearly, for sensors 1 and 2, the relationship between temperature changes and wavelength shifts were quite similar. In detail, sensors 1 and 2 were bonded to the same material (aluminum); hence, the thermal expansion coefficients were the same for both. Although the thicknesses of the plates to which Sensor 1 and Sensor 2 were bonded were different (2 mm and 10 mm, respectively), the stretch ratios caused by the bonded material under the same temperature variations were more or less the same, signaling no dependency in the temperature response on the thickness of the material. On the other hand, Sensor 3 is left free in the air, the thermal expansion is not so steady compared with metal materials, which has been verified by the experimental results shown in Figure 13d.

**Figure 14:**
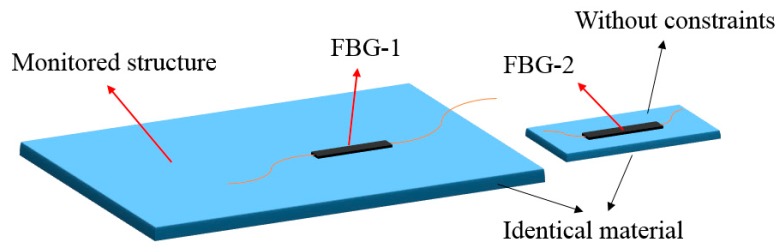
Configuration of the temperature compensation method.

Since the temperature variations can make a great difference to the measurement data, the temperature effect must be compensated for in order to obtain the true strain values caused by mechanical force. To eliminate the temperature-induced effect from strain measurement data, an additional FBG sensor (called the dummy sensor) which is isolated from mechanical force, was kept in the same environment. This method can be straightforwardly illustrated in Figure 14. As shown in Figure 14, FBG-1 was attached on the monitored structure, and the dummy sensor FBG-2 was bonded to a plate with the identical material of the monitored structure. The strain ε induced by mechanical force can be calculated as follows:

(3) $$\varepsilon =\frac{\Delta \lambda_{B-1}-\Delta \lambda_{B-2}}{\lambda_{B-1}\kappa_{\varepsilon -1}}$$

where $\kappa_{\varepsilon -1}$ is the strain sensitivity coefficients of FBG-1, $\Delta \lambda_{B-1}$ is the wavelength drift of FBG-1, and $\Delta \lambda_{B-2}$ is the wavelength drift of FBG-2.

### 3.5. FBG Monitoring System

**Figure 15:**
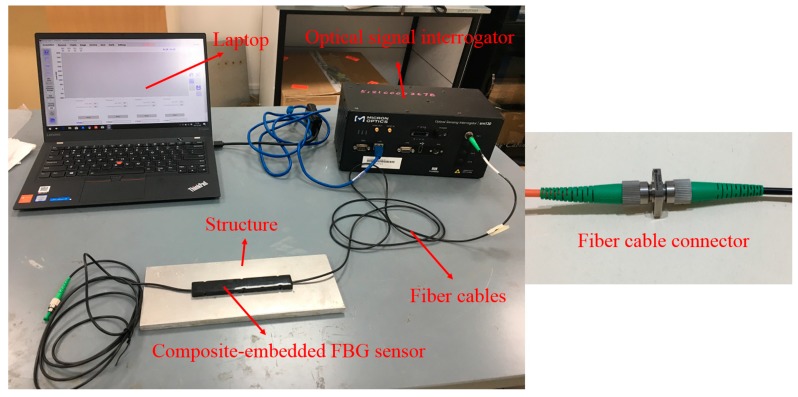
FBG monitoring system based on a composite embedded FBG sensor.

After selecting the suitable FBG sensor for offshore applications, an FBG monitoring system based on the composite embedded sensor is introduced. The FBG monitoring system (as shown in Figure 15) includes composite embedded FBG sensors, protected fiber cables of an appropriate length for signal transmission, fiber cable connectors, an optical signal interrogator, and a human–computer interaction module (laptop or computer).

In the FBG monitoring system, all of the FBG sensors are connected with each other by using fiber connectors, and signals from all of the sensors can be obtained by just one fiber. The length of the fiber cables depends on the distance among the measurement points; sometimes, it can reach hundreds of meters. The composite embedded FBG sensors can be bonded using resin-based epoxy during installation; no welding and drilling is required. Thus, the installation method is convenient, reliable, and harmless to the offshore structures.

## 4. Experimental Design and Results Based on an FPSO Model

In order to test the practicality and accuracy of the proposed FBG monitoring system, it is employed to measure the strain level on the surface plate of an FPSO model. The FPSOs, which are large in size and highly technical, have been widely constructed for offshore oil and gas fields [40]. Generally, the FPSO operates at a specific location, and it is unlikely for them to avoid adverse weather conditions. Therefore, the FPSO needs a very reliable and effective SHM system in order to monitor the structural strength and detect fatal fatigue damage during the service period [41]. The structural fatigue assessment is of vital importance, while the prerequisite for structural fatigue analysis is the strain data under different working conditions.

**Figure 16:**
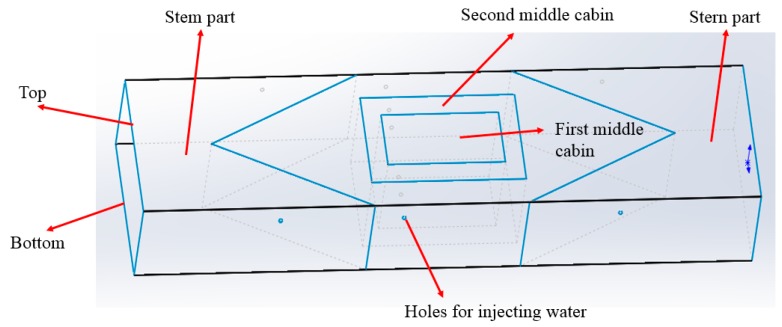
Design drawing of the floating, production, storage, and offloading unit (FPSO) model.

In this section, laboratory experiments are done to test the performance of the FBG monitoring system to make sure it can record valuable and accurate data. The experiments are carried out on a 1/150 scale FPSO model that is made of acrylate plates with a thickness of 2 mm. Since it is difficult and unnecessary to build all of the details similar to the real FPSO, only the main structures are featured when constructing the small FPSO model. The design drawing of the FPSO model with a size of 2000 mm × 450 mm × 250 mm is shown in Figure 16.

During its service life, an FPSO is predominantly loaded by still water bending moment (SWBM) and vertical wave-induced bending moment (VWBM). In the laboratory tests, we only simulated the SWBM, which depends basically on the longitudinal distribution of the self-weight, the cargo, or the deadweight. Two working conditions, the full load condition and the ballast draft condition, were considered based on the FPSO model. The proposed FBG monitoring system was used to detect the strain values of the FPSO model for the two working conditions.

**Figure 17:**
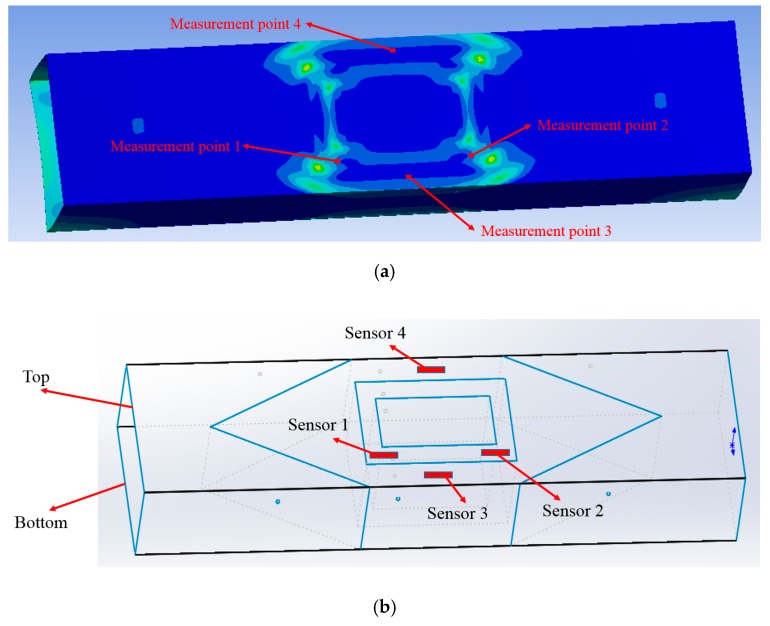
(**a**) Strain distribution under a certain working condition; (**b**) Arrangement of FBG sensors.

In order to locate the measurement points (hot spots) when monitoring the FPSO model, finite element analysis (FEA) was conducted. According to the simulation results (as shown in Figure 17a), under a certain working condition, four measurement points were determined, and the arrangement of sensors is shown in Figure 17b. Thus, four composite embedded FBG sensors were installed on the surface of the FPSO model. In detail, sensors 1 and 2 were symmetrically placed at the hot points, and sensors 3 and 4 were symmetrically placed at the larboard and starboard. The total length of the composite embedded FBG sensor was 130 mm, and its FBG length was only 10 mm. Since there are many continuous fixed points on the surface of the composite embedded FBG sensor, the sensor span can be from 10 mm to 120 mm. The measured strain from the FBG sensor is the average strain on the sensor span. In the experiment, the chosen hot spots were set to have a length of 15 mm, and the span of the sensors’ fixing points was also set to 15 mm. To verify the FBG sensor system and calculate the average strain conveniently, it is desired that the strain values are relatively high, but the strain variations along the sensor span are not drastic at the measurement points.

**Figure 18:**
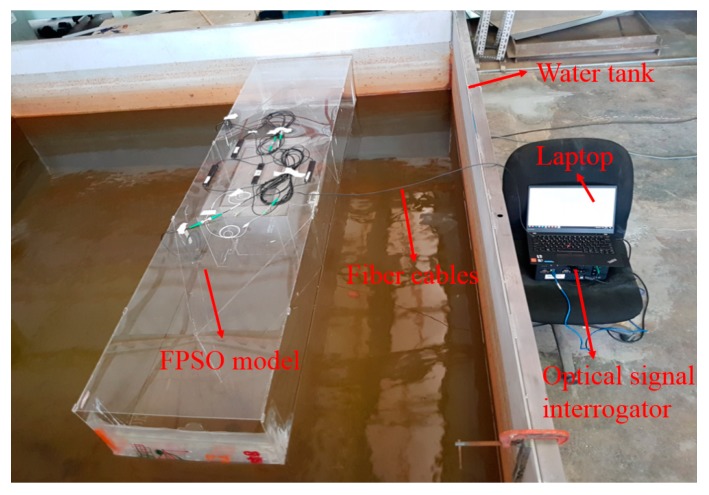
The experimental scene in the laboratory.

To simulate the full load condition and ballast draft condition, the FPSO model was put into a water tank with a length of 2.3 m and a width of 2.3 m (as shown in Figure 18). Since the experiments are carried out in the laboratory, the environment temperature is very steady, and temperature compensation is thus not needed.

The full loading working condition can be realized by injecting water into the first middle cabin and second middle cabin. The ballast draft condition can be realized by injecting water into the stem part and stern part. The detailed procedures are described as follows.

(1) Experiment 1: The FPSO model is put in the water tank for 15 minutes to let it reach the steady state. The water is injected into the first middle cabin in two-liter steps until it is full. The wavelengths of the four FBG sensors are recorded at every step. Further, the water is injected into the second middle cabin in two-liter steps until it is full. Again, the wavelengths of the four FBG sensors are recorded at every step.
(2) Experiment 2: The FPSO model is put in the water tank for 15 minutes to let it reach the steady state. The water is injected into both the stem part and stern part in two-liter steps until the total amount of the injected water reaches 30 liters, and the wavelengths of the four FBG sensors are recorded at every step.

**Figure 19:**
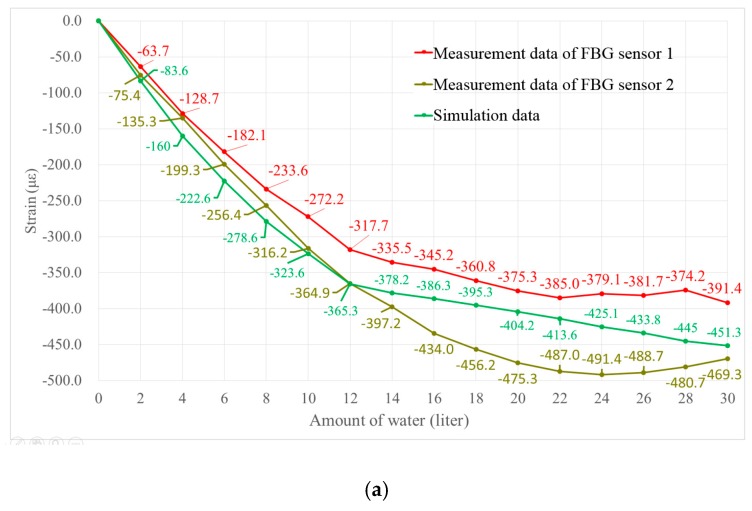

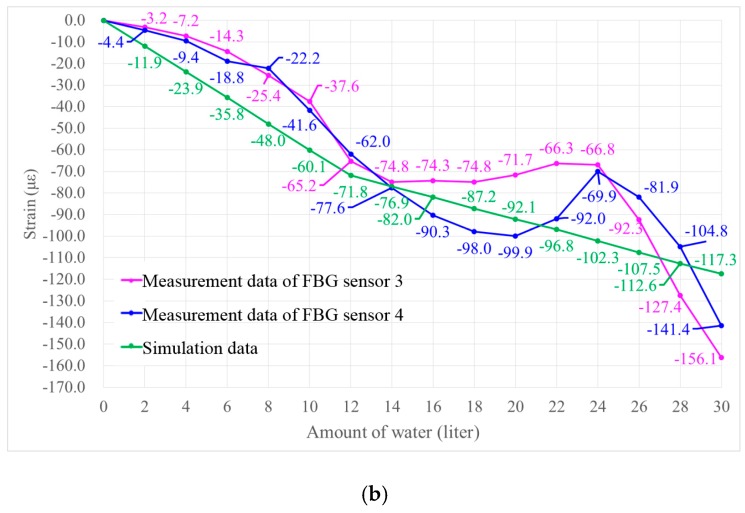
Comparison of measurement data and simulation data under full load condition. (**a**) For sensors 1 and 2; (**b**) For sensors 3 and 4.

**Figure 20:**
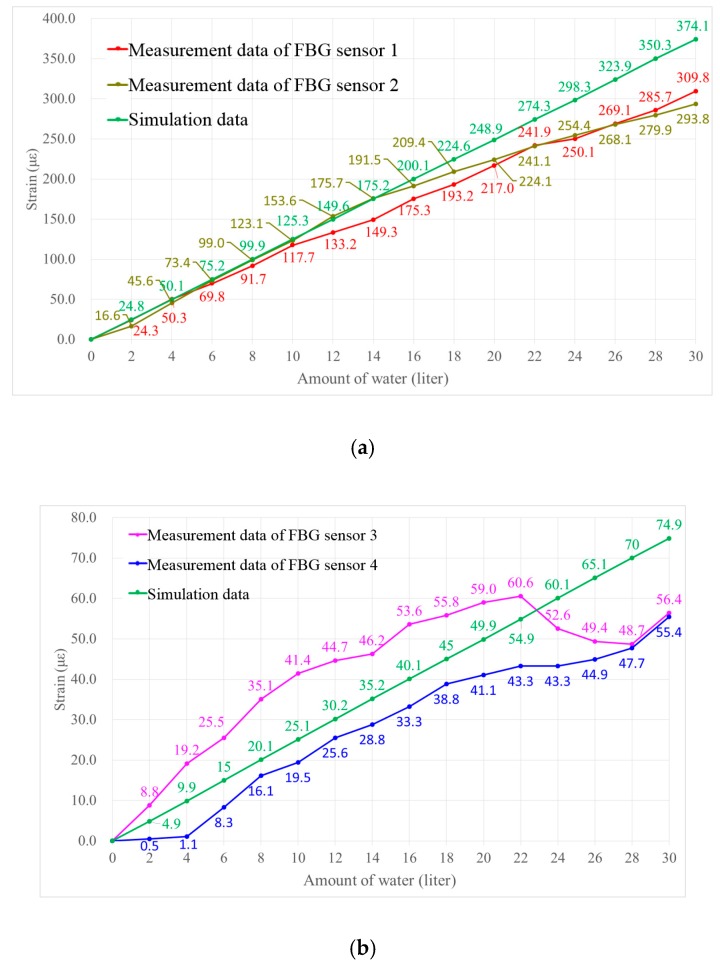
Comparison of measurement data and simulation data under ballast draft condition. (**a**) For sensors 1 and 2; (**b**) For sensors 3 and 4.

According to the measurement data obtained by the four FBG sensors, the strain values that were induced due to water injection are plotted in Figure 19 (under full load condition) and in Figure 20 (under ballast draft condition). For comparison, the simulation data obtained by FEA under the same working conditions is also shown. Since the relative positions of sensors 1 and 2 are symmetrical about the transverse cross section, and the relative positions of sensors 3 and 4 are symmetrical about the longitudinal cross section, , the measurement data of sensors 1 and 2 should be the same theoretically, and likewise for sensors 3 and 4.

As shown in Figure 19a and Figure 20a, the measurement data of sensors 1 and 2 have the same trend, and they are consistent with the simulation data. However, the data from sensors 3 and 4 do not match well with the simulation (Figure 19b and Figure 20b). The discrepancies between the experimental and simulation data can be attributed to the imperfections in the FPSO model fabrication. Since the experiments are carried out in water, the 1/150 scale FPSO model must be sufficiently flexible so that considerable strains can be induced. Thus, the FPSO model whose length is 2000 mm is made of acrylate plates with a thickness of 2 mm. Actually, it is quite challenging to keep all of the connection lines in ideal conditions when building the FPSO model. The glue connecting the acrylate plates may fail at some joints, causing the strain to not transfer properly. This caused the differences observed for analyzed cases. However, the variation trends in the measurement data of sensors 3 and 4 are still coincident with that of the simulation data, as shown in Figure 19b and Figure 20b. Overall, the composite embedded FBG sensor and the proposed FBG monitoring system are competent to monitor the strain change on the surface of the FPSO model.

## 5. Conclusions

In this study, we address some practical issues regarding the application of customized FBG sensors for the SHM of offshore structures. A comprehensive study of various available FBG sensors is conducted, and the experimental results suggest that the FBG sensor embedded into composite is the best suited for offshore applications in consideration of its robustness, sensitivity, stability, and repeatability. To test the performance of the composite embedded FBG sensor in the harsh offshore environment, a series of dynamic tests and durability tests are conducted, including experiments on FBG sensors subjected to environments similar to offshore conditions such as strong sunlight, heavy rain, and salty water. An FBG monitoring system based on the composite embedded FBG sensor is then introduced and employed to measure the strain values of an FPSO model under full load and ballast draft conditions. The experimental results show that the composite embedded FBG sensors competently monitor the offshore structures with high practicality and accuracy.

## Figures and Tables

**Table 1:** Strain values under different loads and percentage differences compared with simulation results.

**Sensors**	**Loads and Results**					
	**10 N**		**20 N**		**30 N**	
	**SV (με)**	**PD**	**SV (με)**	**PD**	**SV (με)**	**PD**
Simulation results	31.13	−−	62.26	−−	93.39	−−
Strain gauge	31.02	−0.35%	61.59	−1.08%	91.65	−1.86%
Bare FBG sensor	32.89	6.65%	63.71	2.33%	96.77	3.62%
FBG sensor embedded into polyimide	37.92	21.81%	73.39	17.88%	108.17	15.83%
Athermal FBG sensor	18.55	−40.41%	34.68	−44.30%	49.19	−47.33%
FBG sensor embedded into composite	30.65	−1.54%	62.10	−0.26%	91.94	−1.55%
**Sensors**	**Loads and results**					
	**10 N**		**20 N**		**30 N**	
	**SV (με)**	**PD**	**SV (με)**	**PD**	**SV (με)**	**PD**
Simulation results	124.52	−−	155.65	−−	186.78	−−
Strain gauge	121.77	−2.21%	151.83	−2.45%	182.91	−2.07%
Bare FBG sensor	129.01	3.60%	161.5	3.76%	193.55	3.60%
FBG sensor embedded into polyimide	142.73	14.62%	179.4	15.26%	216.18	15.74%
Athermal FBG sensor	63.71	−48.84%	75.81	−51.29%	88.71	−52.51%
FBG sensor embedded into composite	122.60	−1.54%	153.2	−1.57%	181.50	−2.83%

* SV: strain value; PD: percentage difference.

**Table 2:** Maximum standard deviation in measurements of all of the FBG sensors.

Sensors	Bare FBG Sensor	FBG Sensor Embedded into Polyimide	FBG Sensor Embedded into Composite	Athermal FBG Sensor
Std (με)	1.7	5.4	2	18

## References

[1] Cui X., Wang Y.Y., Zeng S.Z., Zhou D.C., Han B.G., Yu X., Ou J.P. (2017). Numerical analysis on design and application of cement-based sensor for structural health monitoring. J. Intell. Mater. Syst. Struct..

[2] Yi T.H., Huang H.B., Li H.N. (2017). Development of sensor validation methodologies for structural health monitoring: A comprehensive review. Measurement.

[3] Goyal D., Rabla B.S. (2016). The Vibration Monitoring Methods and Signal Processing Techniques for Structural Health Monitoring: A Review. Arch. Comput. Methods Eng..

[4] Pandey A., Arockiarajan A. (2017). An experimental and theoretical fatigue study on macro fiber composite (MFC) under thermo-mechanical loadings. Eur. J. Mech. A Solids.

[5] Yin F., Ye D., Zhu C., Qiu L., Huang Y.A. (2017). Stretchable, Highly Durable Ternary Nanocomposite Strain Sensor for Structural Health Monitoring of Flexible Aircraft. Sensors.

[6] Khuc T., Catbas F.N. (2018). Structural Identification Using Computer Vision-Based Bridge Health Monitoring. J. Struct. Eng..

[7] Chang Z.Y., Yu Y.Q., Qi T.G. (2017). Study on dynamic characteristics of hydraulic pumping unit on offshore platform. China Ocean Eng..

[8] Islam M.R., Bagherifaez M., Ali M.M., Chai H.K., Lim K.S., Ahmad H. (2015). Tilted Fiber Bragg Grating Sensors for Reinforcement Corrosion Measurement in Marine Concrete Structure. IEEE Trans. Instrum. Meas..

[9] Nishimoto K., Fucatu C.H., Masetti I.Q. (2002). Dynasim—A time domain simulator of anchored FPSO. J. Offshore Mech. Arct. Eng..

[10] Nam B.W., Kim Y., Hong S.Y. (2016). Time-domain simulation of berthing problem between FPSO and shuttle tanker in waves. Appl. Ocean Res..

[11] Song G., Gu H., Mo Y.L., Hsu T.T.C., Dhonde H. (2007). Concrete structural health monitoring using embedded piezoceramic transducers. Smart Mater. Struct..

[12] Chen Z.S., Zhou X., Wang X., Dong L.L., Qian Y.H. (2017). Deployment of a Smart Structural Health Monitoring System for Long-Span Arch Bridges: A Review and a Case Study. Sensors.

[13] Li T.L., Tan Y.G., Han X., Zheng K., Zhou Z.D. (2017). Diaphragm Based Fiber Bragg Grating Acceleration Sensor with Temperature Compensation. Sensors.

[14] Li T.L., Tan Y.G., Zhou Z.D. (2017). String-type based two-dimensional fiber Bragg grating vibration sensing principle and structure optimization. Sens. Actuators A Phys..

[15] Tian S.H., Yang Z.B., Chen X.F., Xie Y. (2015). Damage Detection Based on Static Strain Responses Using FBG in a Wind Turbine Blade. Sensors.

[16] Mieloszyk M., Ostachowicz W. (2017). An application of Structural Health Monitoring system based on FBG sensors to offshore wind turbine support structure model. Mar. Struct..

[17] Opoka S., Soman R., Mieloszyk M., Ostachowicz W. (2016). Damage detection and localization method based on a frequency spectrum change in a scaled tripod model with strain rosettes. Mar. Struct..

[18] Yi J.H. (2016). Laboratory tests on local damage detection for jacket-type offshore structures using optical FBG sensors based on statistical approaches. Ocean Eng..

[19] Xu J., Yang D.X., Qin C., Jiang Y.J., Sheng L.X., Jia X.Y., Bai Y., Shen X.H., Wang H.Y., Deng X. (2015). Study and Test of a New Bundle-Structure Riser Stress Monitoring Sensor Based on FBG. Sensors.

[20] Majewska K., Mieloszyk M., Ostachowicz W., Król A. (2014). Experimental method of strain/stress measurements on tall sailing ships using Fibre Bragg Grating sensors. Appl. Ocean Res..

[21] Ren L., Jia Z.G., Ho M.S.C., Yi T.H., Li H.N. (2014). Application of fiber Bragg grating based strain sensor in pipeline vortex-induced vibration measurement. Sci. China Technol. Sci..

[22] Sun A., Farrell G., Semenova Y., Chen B., Li G.Y., Lin Z.Q. (2011). The distributed dynamic combined-stresses measurement of ship thruster inner-skin using fiber Bragg grating sensor rosette array. Optik.

[23] Wu W.H., Wang Y.L., Tang D., Yue Q.J., Du Y., Fan Z.L., Lin Y., Zhang Y.T. (2016). Design, implementation and analysis of full coupled monitoring system of FPSO with soft yoke mooring system. Ocean Eng..

[24] Li H.C.H., Herszberg I., Davis C.E., Mouritz A.P., Galea S.C. (2006). Health monitoring of marine composite structural joints using fiber optic sensors. Compos. Struct..

[25] Ren L., Li H.N., Zhou J., Li D.S., Sun L. (2006). Health monitoring system for offshore platform with fiber Bragg grating sensors. Opt. Eng..

[26] Kim M. (2006). A Smart Health Monitoring System with Application to Welded Structures using Piezoceramic and Fiber Optic Transducers. J. Int. Mater. Syst. Struct..

[27] Hong C.Y., Zhang Y.F., Zhang M.X., Gordon L.L.M., Liu L.Q. (2016). Application of FBG sensors for geotechnical health monitoring, a review of sensor design, implementation methods and packaging techniques. Sens. Actuators A Phys..

[28] Chen W.P., Shih F.H., Tseng P.J., Shao C.H., Chiang C.C. (2015). Application of a Packaged Fiber Bragg Grating Sensor to Outdoor Optical Fiber Cabinets for Environmental Monitoring. IEEE Sens. J..

[29] Wan K. (2014). Quantitative sensitivity analysis of surface attached optical fiber strain sensor. IEEE Sens. J..

[30] Li J., Neumann H., Ramalingam R. (2015). Design, fabrication, and testing of fiber Bragg grating sensors for cryogenic long-range displacement measurement. Cryogenics.

[31] Pachava V.R., Kamineai S., Madhuvarasu S.S., Putha K., Mamidi V.R. (2015). FBG based high sensitive pressure sensor and its low-cost interrogation system with enhanced resolution. Photonic Sens..

[32] Tai K., Hasegawa A., Tomita A. (2003). Embedding optical fibers in metal alloys. Instrum. Meas. Mag. IEEE.

[33] Feng Y., Zhang H., Li Y.L., Rao C.F. (2010). Temperature Sensing of Metal-Coated Fiber Bragg Grating. IEEE/ASME Trans. Mechatron..

[34] Huang J.Y., Van Roosbroeck J., Vlekken J., Daerden E., Martinez A.B., Geernaert T., Berghmans F., Van Hoe B., Lindner E., Caucheteur C. Packaged FBG based optical fiber sensor for simultaneous pressure and temperature monitoring. Proceedings of the Fiber Optic Sensors and Applications XV.

[35] Alemohammad H.R., Foroozmehr E., Cotten B.S., Toyserkani E. (2013). A Dual-Parameter Optical Fiber Sensor for Concurrent Strain and Temperature Measurement: Design, Fabrication, Packaging, and Calibration. J. Lightw. Technol..

[36] Kuang Y., Guo Y., Xiong L., Liu W. (2018). Packaging and Temperature Compensation of Fiber Bragg Grating for Strain Sensing: A Survey. Photonic Sens..

[37] Lau K.T., Yuan L.B., Zhou L.M., Wu J.S., Wang C.H. (2001). Strain monitoring in FRP laminates and concrete beams using FBG sensors. Compos. Struct..

[38] Tian K.K., Liu Y.L., Wang Q.M. (2005). Temperature-independent fiber Bragg grating strain sensor using bimetal cantilever. Opt. Fiber Technol..

[39] Luo D., Li P., Yue Y.C., Ma J.X., Yang H.Z. (2017). In-Fiber Optic Salinity Sensing: A Potential Application for Offshore Concrete Structure Protection. Sensors.

[40] Chen N.Z. (2016). Hull girder reliability assessment for FPSOs. Eng. Struct..

[41] Kim S., Kim M.H. (2015). Dynamic behaviors of conventional SCR and lazy-wave SCR for FPSOs in deepwater. Ocean Eng..

